# 2579. Pneumonia Severity Index and CURB-65 scoring for the evaluation of community associated pneumonia in patients with cancer at a major cancer center

**DOI:** 10.1093/ofid/ofad500.2195

**Published:** 2023-11-27

**Authors:** Varshini Gali, Yeon Joo Lee, Krupa Jani, Anna Kaltsas, Susan K Seo, Melvili Cintron

**Affiliations:** Weill Cornell Medicine, New York, New York; Memorial Sloan Kettering Cancer Center, New York, New York; Memorial Sloan Kettering Cancer Center, New York, New York; Memorial Sloan Kettering Cancer Center, New York, New York; Memorial Sloan Kettering, New York, New York; Memorial Sloan Kettering Cancer Center, New York, New York

## Abstract

**Background:**

Cancer patients with pneumonia (PNA) have 2 to 21 times higher mortality rate compared to the general population. The Pneumonia Severity Index (PSI) and CURB-65 scale have been used to predict mortality for patients with community acquired pneumonia (CAP). While these scales are well studied in the general population, there is a paucity of research on the utility of PSI and CURB-65 for predicting outcomes from CAP in patients with cancer. In this study, we assessed the relationship between PSI and CURB-65 score and 30-day mortality of CAP in cancer patients at Memorial Sloan Kettering Cancer Center (MSKCC).

**Methods:**

A prospective study at MSKCC was conducted between 01/2023 and 06/2023 on patients who presented with clinical symptoms consistent with PNA and had a urine streptococcus antigen test sent for evaluation. PNA was defined as pulmonary infiltrates on chest X-ray or CT < 48 hours of index event and ≥ 1 clinical symptoms (fever, cough, dyspnea, increased sputum production, hypoxemia). Descriptive statistics were performed to report participant outcomes based on PSI and CURB-65 score and to identify the factors associated with CAP in this population. PSI and CURB-65 scores were derived as described in the literature, based on patient comorbidities (including neoplasia), clinical signs and laboratory values. Higher score is associated with worsening 30-day mortality.

**Results:**

For 86 participants with who presented with symptoms of PNA and had a urine antigen test sent, 43 (50%) were diagnosed with CAP (Figure 1). The median age was 70 (range: 29-88), 21 (49%) were male, and 16 (37%) had hematologic malignancies (Table 1). Etiologies of CAP were identified in 8 patients (bacteria in 4 patients, virus in 3 patients, fungus in 1 patient). Of the 28 patients with 30-day outcome data available, 5 (18%) died within 30 days of CAP diagnosis. All 5 deceased participants were categorized as PSI Class IV or V (moderate to high risk); however, these participants only scored 0-2 (low to moderate risk) on the CURB-65 scale (Table 2).

**Figure:**
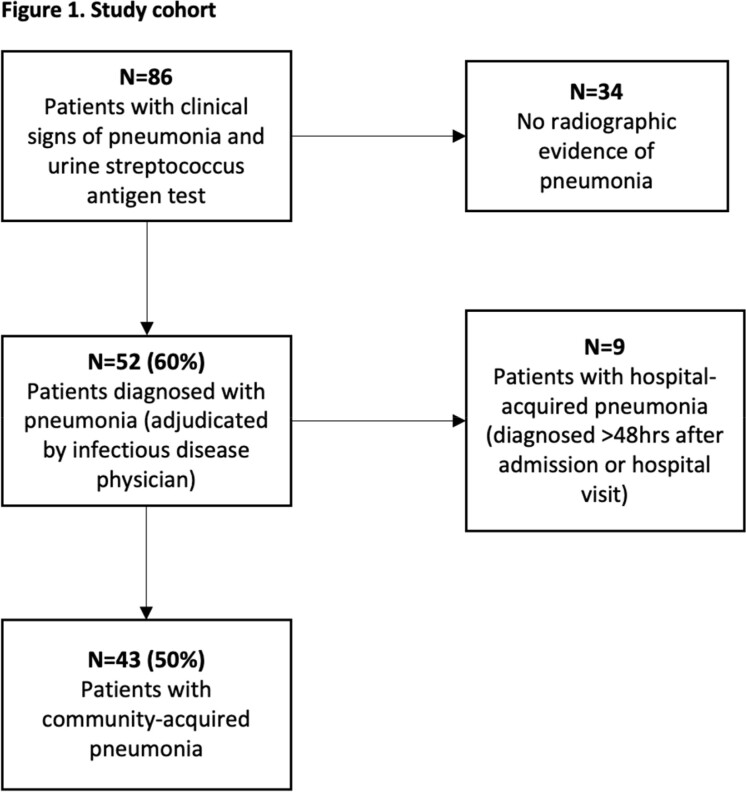

**Figure:**
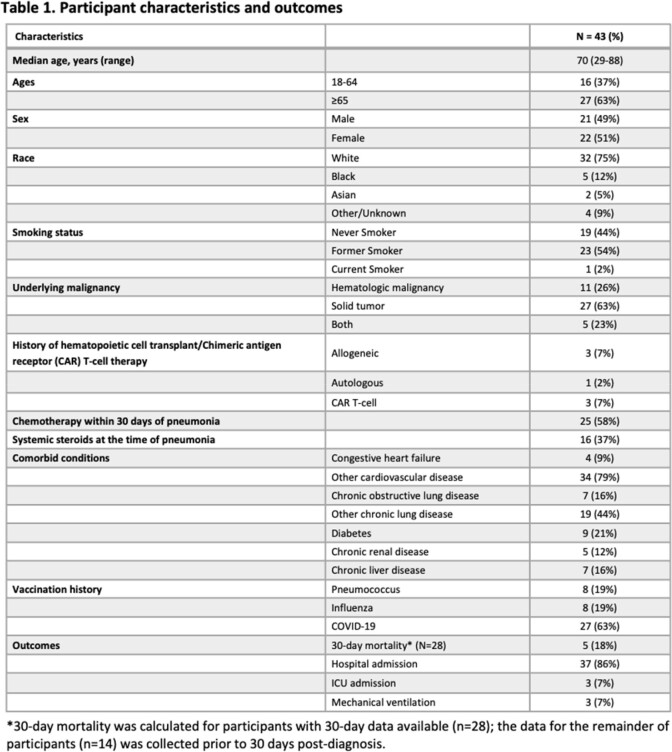

**Figure:**
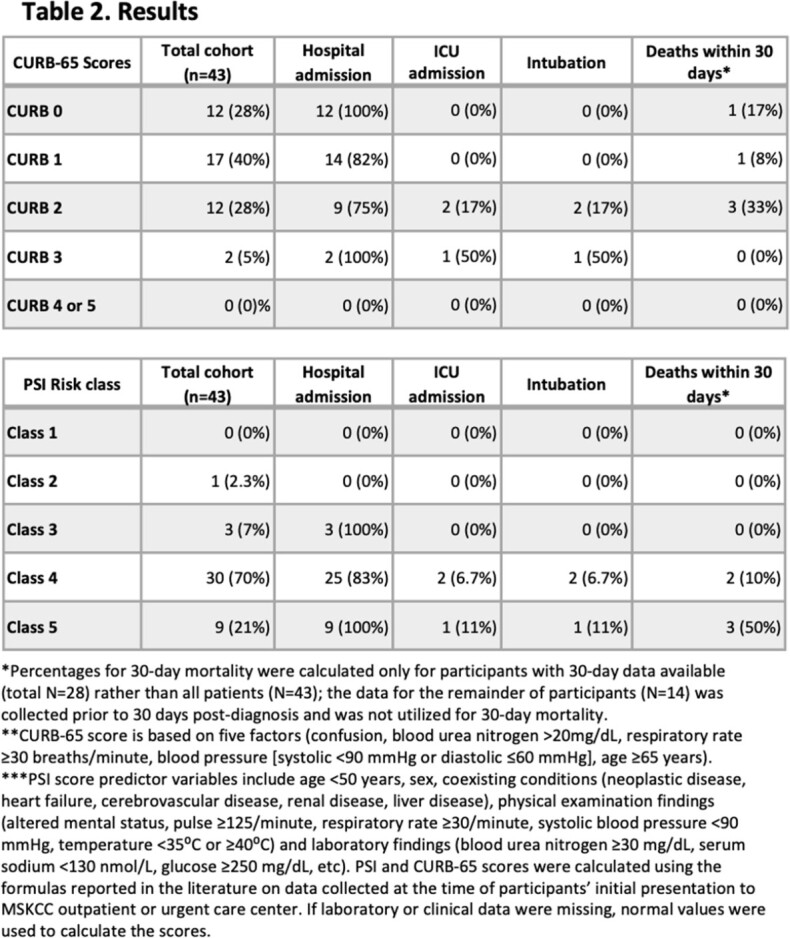

**Conclusion:**

We observed a 30-day overall mortality (n=28) of 18%. For cancer patients who present with CAP, PSI class provides a better means to assess mortality risk than CURB-65 score. Further study is needed to validate our findings.

**Disclosures:**

**Yeon Joo Lee, MD, MPH**, AiCuris: institutional research support for clinical trials|Karius: institutional research support for clinical trials|Merck: Grant/Research Support|Scynexis: institutional research support for clinical trials **Susan K. Seo, MD**, Merck: Grant/Research Support

